# Considerations for epidemiological studies investigating emerging post-acute infection syndromes: Long Covid as a case study

**DOI:** 10.1016/j.eclinm.2026.103833

**Published:** 2026-03-16

**Authors:** Daniel Ayoubkhani, Christina J. Atchison, Amitava Banerjee, Chris Brightling, Melanie Calvert, Ian Diamond, Rosalind M. Eggo, Paul Elliott, Rachael A. Evans, Shamil Haroon, Emily Herrett, Vahé Nafilyan, Lauren L. O'Mahoney, Snehal M. Pinto Pereira, Ash Routen, Roz Shafran, Terence Stephenson, Jonathan Sterne, Helen Ward, Francesco Zaccardi, Kamlesh Khunti

**Affiliations:** a Diabetes Research Centre, University of Leicester, Leicester, UK; b Office for National Statistics, Newport, UK; c School of Public Health, Imperial College London, London, UK; d Institute of Health Informatics, University College London, London, UK; e Barts Heart Centre, Barts Health NHS Trust, London, UK; f Institute for Lung Health, National Institute for Health and Care Research Leicester Biomedical Research Centre, Department of Respiratory Science, University of Leicester, Leicester, UK; g Centre for Patient-Reported Outcomes Research, University of Birmingham, Birmingham, UK; h National Institute for Health and Care Research Birmingham Biomedical Research Centre, University of Birmingham, Birmingham, UK; i National Institute for Health and Care Research Applied Research Collaboration, West Midlands, University of Birmingham, Birmingham, UK; j Former National Statistician, Government Statistical Service, Newport, UK; k Faculty of Epidemiology and Population Health, London School of Hygiene & Tropical Medicine, London, UK; l Department of Applied Health Sciences, University of Birmingham, Birmingham, UK; m Department of Targeted Intervention, Division of Surgery and Interventional Science, University College London, London, UK; n University College London Great Ormond Street Institute of Child Health, London, UK; o Bristol Medical School, University of Bristol, Bristol, UK; p National Institute for Health and Care Research Bristol Biomedical Research Centre, Bristol, UK; q Health Data Research UK South-West, UK

**Keywords:** Long Covid, Post-COVID-19 condition, Post-acute infection syndromes, PAIS, Methodology, Epidemiology

## Abstract

Epidemiological research studies into Long Covid, currently defined by prolonged symptoms after SARS-CoV-2 infection, have reported widely varying prevalence estimates. As well as rapidly evolving scientific knowledge of Long Covid, these differences are partly driven by substantial methodological heterogeneity between studies, including the outcome definition of Long Covid; duration of follow-up; study design, period and population; sampling frame; data source; and the statistical techniques employed. Having a robust understanding of the prevalence of and risk factors for Long Covid is essential for informing treatment pathways, service provision and policy decisions. In preparation for the public health response to future epidemics and pandemics, this review outlines key epidemiological and statistical considerations and recommendations when designing studies of emerging post-acute infection syndromes, focussing on Long Covid as a case study.

## Introduction

A proportion of people infected with SARS-CoV-2 experience symptoms persisting for months or years beyond the acute infection, known as Long Covid, Post-COVID-19 Condition or Post-COVID-19 Syndrome. Symptoms can be wide-ranging and multi-system in nature,^1^ and may have deleterious impacts on quality of life^2^ and the ability to work.^3^ Chronic illness is well recognised following acute infection by a range of viral pathogens.^4^ For example, post-acute infection syndromes (PAISs) have been recorded following the acute phases of Ebola,^5^, ^6^, ^7^, ^8^, ^9^, ^10^, ^11^ chikungunya,^12^, ^13^, ^14^, ^15^, ^16^ West Nile fever,^17^^,^^18^ and dengue fever.^19^, ^20^, ^21^ Perhaps most directly relevant to Long Covid is post-SARS syndrome, caused by the SARS-CoV-1 virus which has a 79% genetic overlap with SARS-CoV-2.^22^ Indeed, the most commonly reported symptoms of post-SARS are strikingly similar to those that characterise Long Covid: fatigue,^23^ myalgia and muscle weakness,^23^^,^^24^ chronic pain,^23^^,^^25^ sleep disturbance,^23^ cognitive dysfunction,^26^ psychological sequalae such as anxiety, depression and post-traumatic stress,^23^^,^^25^, ^26^, ^27^ functional impairment,^24^^,^^25^ and reduced general health status^28^ and quality of life.^27^

However, PAISs have historically been under-researched, with epidemiological studies typically comprising small sample sizes, non-generalisable study populations, and limited follow-up. The scale of SARS-CoV-2 infections, with over 765 million cases globally by the end of the pandemic,^29^ means a very large number of people are likely to have been affected by Long Covid within a relatively short period of time. For example, one study has estimated a cumulative global incidence of Long Covid of 400 million by the end of 2023.^30^ Therefore, epidemiological research into Long Covid presents an opportunity to improve scientific understanding of other PAISs, and thus the health and wellbeing of individuals experiencing these conditions.

Across the extensive epidemiological research into Long Covid, prevalence estimates span nearly the entire range of numerically possible values, from 0%^31^ to 96%^32^ of SARS-CoV-2 infections. As well as rapidly evolving scientific knowledge of Long Covid, these differences are partly driven by substantial methodological heterogeneity between studies,^33^ including the choice of data source, study population and design, exposure and outcome definitions, and statistical techniques. Having a robust understanding of the prevalence of and risk factors for Long Covid is essential for informing pathophysiological mechanisms, treatment pathways, healthcare service provision, and policy and spending decisions.

Drawing on the combined experiences of a group of UK researchers across various nationally funded studies of Long Covid,^34^ this review outlines key epidemiological and statistical considerations when designing studies of emerging PAISs, focussing on Long Covid as a case study. These design considerations are summarised in Table 1. In preparation for the public health response to future epidemics and pandemics, the review concludes with recommendations for epidemiological research into chronic disease following an acute infection with a novel pathogen.

**Table 1:** Advantages and disadvantages of key design options for epidemiological studies of Long Covid.

Design aspect	Design option	Advantages	Disadvantages
Choice of outcome definition	Clinical case definitions Specific symptoms Self-reported Long Covid Self-reported recovery	• Nationally/internationally agreed standards • Facilitates clinical relevance of research outputs • Provides potentially useful descriptive information on individual symptoms (e.g. duration, severity, impact) • Facilitates identification of symptom clusters, which may have utility for hypothesis generation and guiding clinical resource use • Reflects participants own lived experience • No dependency on pre-defined symptom lists • Avoids reliance on pre-defined symptom lists and understanding of the term ‘Long Covid’ • Has discriminatory ability in terms of symptom quantity/burden, physical function, exercise capacity, and health-related quality of life	• No requirement for previous SARS-CoV-2 test may make it more difficult to attribute causality • Exclusion of other conditions may be difficult to operationalise in epidemiological studies • Overall Long Covid prevalence estimate dependent on symptoms included in the list • Estimate may lack specificity as many Long Covid symptoms are common in the general population at any given time, particularly if ‘any one symptom’ definition is used • Requires individuals to correctly attribute their symptoms to past SARS-CoV-2 infection • Dependent on understanding of terminology • Requires individuals to correctly attribute their health status to past SARS-CoV-2 infection • Not necessarily an indicator of the presence of Long Covid
Duration and nature of follow-up	Single timepoint Multiple timepoints Retrospective data collection Prospective data collection	• Certain timepoints, e.g. 12 weeks post-infection, have clinical relevance • Allows for inferences regarding improvement or deterioration in symptoms over time • Possible to perform within-person analysis of symptom duration, relapse, remission and recovery if data are collected longitudinally • Possible to control for pre-infection symptomatology if longitudinal data are collected prior to infection • Mitigates against recall bias if participants are asked about their current symptoms	• Does not allow for inferences regarding improvement or deterioration in symptoms • Longitudinal data collection can be challenging and expensive to operationalise • Potential for recall bias if participants systematically misestimate symptom duration
Use and choice of control groups	Uncontrolled study design Controlled study design ‘Never positive’ control group ‘Always negative’ control group Historical controls Contemporaneous controls	• May facilitate more robust inferences about quantities such as attributable risk and lead to stronger aetiological interpretations • Does not require data on negative test results, which may not be available • Controls for differential test-seeking behaviours according to attributes potentially related to Long Covid risk • No risk of the pool of non-exposed controls becoming depleted over time as infection spreads throughout the population • No risk of confounding by temporal effects	• Symptom prevalence in previously infected participants not a reliable measure of Long Covid if symptoms are common in general population • Identifying non-infected controls became challenging as SARS-CoV-2 spread • People who never test, irrespective of symptoms, may be systematically different to those who test regularly in ways related to Long Covid risk • People who repeatedly tested negative in community testing programmes may have other underlying health conditions causing symptoms, so may not be a representative control group • Studies with random/routine testing, irrespective of symptoms and healthcare-seeking behaviours, are expensive to operationalise • Comparisons between exposed and non-exposed groups are fully confounded by temporal effects • The pool of non-exposed controls may become depleted over time
Choice of study population	General population-based sample People with a recorded SARS-CoV-2 test in community testing programmes People previously admitted to hospital with acute COVID-19	• Allows inferences on Long Covid prevalence to be generalised to the broader general population • Possible to make use of routinely collected data • Possible to make use of routinely collected data	• Logistically challenging and relatively expensive to operationalise • Many people likely to have sought a test due to experiencing symptoms, so resulting Long Covid prevalence estimates may not be generalisable beyond people with acute-phase illness • Certain population subgroups may be underrepresented in community testing data due to differential test-seeking behaviours and access • Long Covid prevalence estimates may be subject to survivorship bias given the relatively high mortality rate among hospital patients
Electronic health records (EHRs) versus sample survey data	EHR databases Sample survey data	• Typically very large datasets, leading to estimates with high statistical precision • Estimating Long Covid prevalence from coded diagnoses will have high positive predictive value, as clinicians must exclude other aetiologies • Facilitate large-scale studies into the real-world effectiveness of therapeutics (e.g. COVID-19 vaccines or treatments for acute COVID-19, such as Paxlovid and Remdesivir) in reducing the risk of Long Covid • Probability sampling guards against selection effects inherent in some EHR datasets	• Much symptom-related information may be held in free-text entries inaccessible to researchers • Estimating Long Covid prevalence from coded cases of Long Covid will have low sensitivity, as patients may experience difficulties in receiving a diagnosis and accessing services • Certain population subgroups may be underrepresented in EHR data due to gradients in healthcare access • Logistically challenging and relatively expensive to operationalise • May be affected by non-response bias, and non-probability samples prone to selection effects • Response bias may be present, and Long Covid prevalence estimates may be sensitive to choice of data collection mode

## Choice of outcome definition

The first descriptions and definitions of Long Covid came from patients,^35^ and individuals with lived experience of the illness have been highly proactive in engaging with research.^36^ Various clinical case definitions of Long Covid have been implemented since then^37^, ^38^, ^39^, ^40^ (Table 2), but these may not be appropriate for research purposes. For example, individuals without a positive test for SARS-CoV-2 should not be excluded from accessing healthcare services for Long Covid, whereas restriction to participants with a test-confirmed history of infection may provide stronger evidence of the aetiology of disease in epidemiological studies. However, this can affect sample sizes and generalisability, with electronic healthcare record (EHR) studies finding 59% of GP-recorded Long Covid patients did not have a positive test for SARS-CoV-2 more than 12 weeks before diagnosis.^41^ This was particularly so during the early stages of the pandemic, before mass community testing for, and surveillance studies of, SARS-CoV-2 were introduced. In one population-based study, just 5% of participants with SARS-CoV-2 antibodies in June–July 2020 had previously received a positive polymerase chain reaction (PCR) test result.^42^ Individuals infected early in the pandemic are therefore likely to be underrepresented in epidemiological studies of Long Covid that require a test-confirmed history of infection, as too are population subgroups with a lower propensity to access and engage with SARS-CoV-2 testing programmes.^43^

**Table 2:** Key characteristics of clinical case definitions of Long Covid in adults.

Source	Term	Duration after infection	Requirement for subsequent continuous persistence of symptoms?	Requirement for limitation to daily functioning?	Requirement for positive SARS-CoV-2 test?	Requirement to rule out other health conditions?
National Institute for Health and Care Excellence	Ongoing Symptomatic COVID-19	4–12 weeks	No	No	No	Yes
	Post-COVID-19 Syndrome	≥12 weeks	No	No	No	Yes
World Health Organization	Long Covid or Post COVID-19 Condition	≥3 months	Yes, ≥2 months	Yes	No	Yes
Centres for Disease Control and Prevention	Post-COVID Conditions	≥4 weeks	No	No	No	No
National Academies of Sciences, Engineering, and Medicine Committee	Long Covid	>3 months	No	No	No	No

Because there is no diagnostic test for Long Covid, clinical case definitions essentially render Long Covid a “diagnosis of exclusion”. This exclusionary requirement is difficult to operationalise in epidemiological research, particularly for large population-based studies which may not have access to the requisite clinical resources (though it has been done in smaller-scale studies in clinical settings^44^^,^^45^). These considerations highlight the need for a research definition of Long Covid, distinct from clinical case definitions, such as that produced by the Children and Young People with Long Covid (CLoCk) study in the UK.^46^ However, five years after the COVID-19 pandemic began, a research definition with population applicability and international consensus still remains elusive.

In the absence of a standardised research definition, some studies have inferred Long Covid from the presence of pre-defined symptoms following SARS-CoV-2 infection.^47^ Over 200 individual symptoms have been implicated in Long Covid,^1^^,^^48^^,^^49^ so prevalence estimates obtained using this approach depend on the symptom list used. This approach may also be unable to discriminate the *signal* of Long Covid from the *noise* of background symptomatology. For example, while symptoms such as anosmia and parosmia are highly specific to SARS-CoV-2, over 15% of the population may experience headache on any particular day,^50^ while fatigue lasting less than six months may affect over 20% of people.^51^ Therefore, defining Long Covid as the presence of at least one of a long list of symptoms, over a potentially long period, may capture most people who truly have the disease (high sensitivity); but also many people who do not (low specificity).

Long Covid has been described as an episodic or fluctuating health condition,^48^^,^^52^, ^53^, ^54^ so a nuanced approach that reflects the frequency with which symptoms occur and resolve is likely to have greater specificity. Several studies have used clustering techniques to identify possible Long Covid phenotype. However, the identified clusters vary and range in number from two^55^, ^56^, ^57^ to 13,^58^ casting doubt on their underlying biological basis and clinical utility, and suggesting dependence on the chosen analytic strategy, study population and sample size. This highlights the need for large-scale epidemiological studies that include biological investigations (such as the ‘Real-time Assessment of Community Transmission’ (REACT) study^59^) and mechanistic work (such as the ‘Post-Hospitalisation COVID-19’ [PHOSP-COVID] study^60^), to shed light on the pathogenesis underpinning symptom clusters.

**Fig. 1:**
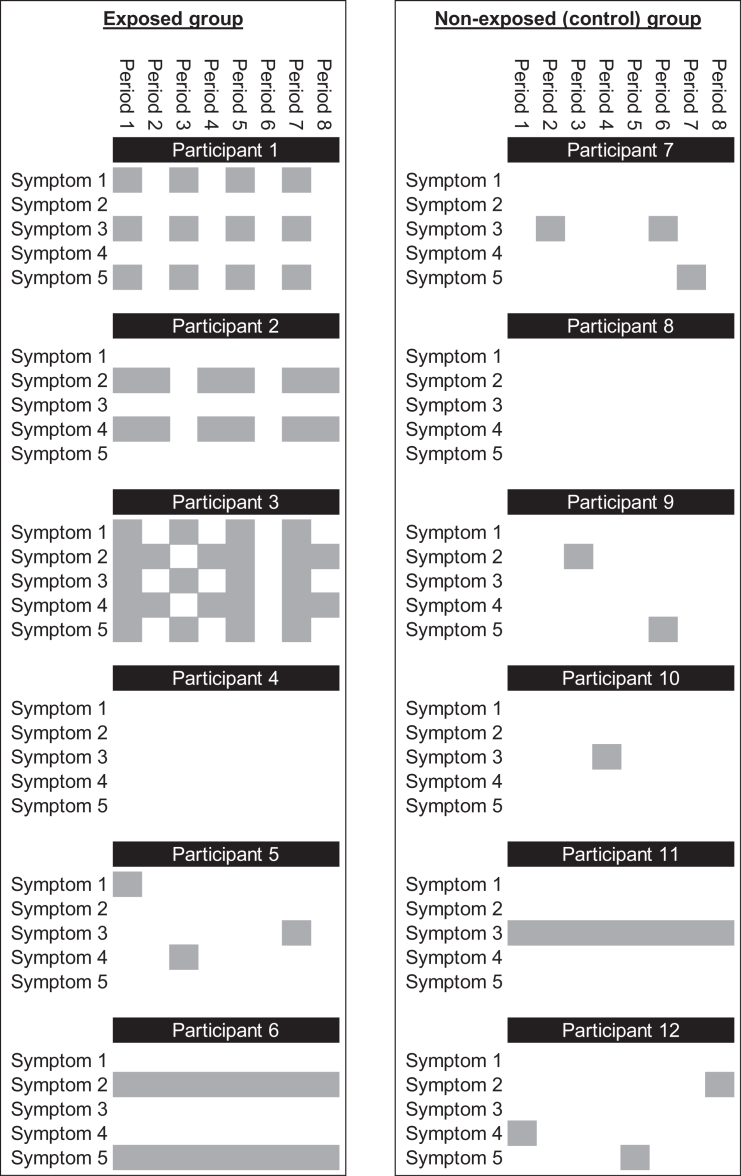
Illustrative example comparing different outcome definitions between exposed (infected) and non-exposed (non-infected) groups. The figure shows symptom trajectories for 12 participants of a fictional study in which 5 symptoms are recorded at 8 follow-up assessments. Grey boxes illustrate when symptoms are present. Participant 1 reports that three symptoms co-occur in every other follow-up period. Participant 2 reports that two symptoms co-occur, with remission in every third follow-up period. Participant 3 reports both of these temporal clusters. Participants 5, 7, 9, 10 and 12 occasionally report individual symptoms (e.g. due to short-term illnesses). Participants 6 and 11 report certain symptoms continuously (e.g. due to pre-existing health conditions). Participants 4 and 8 do not report any symptoms during follow-up. The outcome ‘at least one symptom reported at least once during follow-up’ is experienced by 5/6 participants in both groups. The outcome ‘at least one symptom reported continuously during follow-up’ is experienced by 1/6 participants in both groups. However, the two temporal symptom clusters are only experienced by participants in the exposed group.

Longitudinal symptom clustering may identify constellations of symptoms that co-occur together over time following infection, reflecting the waxing and waning of illness reported by people with lived experience. This family of approaches includes model-based, algorithmic and functional clustering methods^61^ and aims to elicit patters in repeated measurements over time, thus reflecting the temporal as well as the cross-sectional characteristics of disease phenotypes. An illustrative example is depicted in Fig. 1: the outcome ‘at least one symptom reported at least once during follow-up’ is experienced by 5/6 participants in both the exposed (infected with SARS-CoV-2) and non-exposed groups, while ‘at least one symptom reported continuously during follow-up’ is experienced by 1/6 participants in both groups. Thus, both these outcomes lack specificity. However, the two temporal symptom clusters, reflecting two potential Long Covid phenotypes, are only experienced by participants in the exposed group, demonstrating the utility of longitudinal symptom data coupled with appropriate clustering techniques. Longitudinal disease clustering has increasingly been used to identify patterns in multimorbidity since before the onset of the pandemic,^62^ though it remains a nascent area of research^63^ and has been under-utilised in Long Covid studies. This is perhaps partly due to the requirement for high-frequency longitudinal data, of which there is a paucity in prospective studies of Long Covid, though attempts have been made to apply longitudinal clustering techniques to symptoms recorded in EHRs.^64^

An alternative to inferring the presence of Long Covid from symptomatology is to allow study participants to self-report their Long Covid status, an approach adopted in official prevalence estimates in the UK^65^ and the US.^66^ This does not require a pre-defined symptom list and makes no *a priori* assumptions over what constitutes Long Covid, favouring individual experiences and perceptions. It may also be useful for assessing (possibly latent) healthcare service demand, as individuals who believe they are experiencing Long Covid may be the ones who are most likely to eventually present to primary care seeking health-related support.

However, under the self-classification approach, it is insufficient for participants to solely identify and report their symptoms; attribution to a past SARS-CoV-2 infection is also required. This relies on participants correctly identifying the aetiology of symptoms, which may have been particularly difficult for people infected early in the pandemic, when large-scale testing was not available and public understanding of COVID-19 was in its infancy; or since the end of the pandemic, with fewer people routinely testing for SARS-CoV-2. Attribution is more difficult with increasing time since the initial infection, as people may develop other illnesses. It may be challenging for people with pre-existing, long-term health conditions to distinguish a new disease from worsening of an existing one.^67^

Furthermore, understanding of the term ‘Long Covid’ varies between people,^68^ and may be limited among people whose first language is not native^69^ and those from countries where Long Covid has received relatively little attention.^70^ This could introduce bias into analyses exploring national or ethnic inequalities in Long Covid. It is impossible to validate the reliability of self-reported Long Covid status against a “gold standard” due to the absence of a diagnostic test and the quality of diagnoses and symptom recording in primary and secondary care.

Studies such as PHOSP-COVID and REACT asked whether participants felt they had fully or partially recovered from a previous SARS-CoV-2 infection. This approach avoids reliance on pre-defined symptom lists and differential understanding of the term ‘Long Covid’. Patient-perceived recovery has been found to have discriminatory ability in terms of symptom quantity, symptom burden using symptom-specific validated measures, physical function, exercise capacity, and health-related quality of life.^71^ However, like the self-classification approach, asking participants about the extent of their recovery assumes that they can correctly attribute current symptoms to a past SARS-CoV-2 infection.

The empirical impact of variation in Long Covid outcome definitions on prevalence estimates has been quantified by a population-based study in the US, in which 20.9% of respondents reported incomplete recovery ≥30 days after SARS-CoV-2 infection; 15.8% reported new, persistent or worsening symptoms; and 4.9% reported activity-limiting symptoms for which they sought healthcare services.^72^

Although a consensus definition is lacking, standardised instruments for assessing *outcomes* of Long Covid do exist. For example, an internationally agreed core outcome set comprising 11 measures has been developed via a modified Delphi process^73^ (though this was not published until over two years into the pandemic, and consensus was not reached on several outcomes). The 2020 PHOSP-COVID study^74^ included a bespoke Patient Symptom Questionnaire comprising over 60 symptoms, five symptom visual analogue scales, and questions on occupational change and lifestyle, alongside a suite of existing validated questionnaires. The COVID-19 Yorkshire Rehabilitation Scale^75^ (C19-YRS) was rapidly deployed in routine clinical practice in 2020 with retrospective validation work, while the Symptom Burden Questionnaire™-Long COVID^76^ (SBQ™-LC) was developed in accordance with guidance from the U.S. Food and Drug Administration (FDA) for use in research settings including clinical trials (now licenced in over 60 countries). Such measures may be used in cost-benefit analysis of interventions (including pharmaceuticals and rehabilitation), health service evaluation, and to assess long-term impacts of disease in a standardised way.^77^

## Duration and nature of follow-up

Evaluating Long Covid at a single timepoint (e.g. 12 weeks post-infection) has clinical relevance,^37^^,^^38^ but more valuable insights can be obtained by describing the percentage of participants reporting symptoms at multiple time points^57^ (ideally both before and after SARS-CoV-2 infection), taking time-varying averages of symptom scores,^78^ or modelling Long Covid status as a function of time since infection using linear regression^79^ or spline fits.^80^ While the proportion of people with residual symptoms appears to fall quite quickly over the first four weeks after infection, and to a lesser extent between four and 12 weeks,^57^ a small number of studies with longer follow-up have demonstrated that the potential for recovery between six months and two years post-infection is more limited.^81^^,^^82^

Long Covid trajectories may be inferred from cross-sectional data if the dataset includes people with varying durations of illness at the time of data collection. However, longitudinal data facilitate within-person analysis of symptom duration, as well as instances of relapse, remission, and recovery. This has been done by both describing proportions of people with new versus pre-existing symptoms at various follow-up times^82^^,^^83^ and using more sophisticated survival analysis techniques to estimate the probability of experiencing continuous symptoms at different timepoints^80^ (albeit through somewhat arbitrary definitions of symptom discontinuation). If longitudinal symptom assessments commence prior to SARS-CoV-2 infection, the researcher may statistically control for pre-infection symptomatology, for example by employing a self-controlled case series design.

Regardless of whether Long Covid status is assessed at a single timepoint or longitudinally, recall bias will arise if the study data are collected retrospectively (particularly after a long period of time) and participants systematically over- or under-estimate the duration of their symptoms. For example, participants may be asked to recall whether they were experiencing symptoms 12 weeks post-infection, or those currently experiencing Long Covid symptoms might be asked to recall when their symptoms first began, possibly several years prior. There is evidence that people with chronic health conditions tend to overestimate their pre-disease quality of health.^84^

## Use and choice of control groups

The role of control (comparator, non-exposed) groups has received substantial attention in Long Covid discourse.^85^, ^86^, ^87^ Control groups may facilitate more robust inferences about quantities such as attributable risk and lead to stronger aetiological interpretations (subject to the usual conditions for causal inference, particularly sufficient control of confounding factors). However, one systematic review found that control groups were used in only 22 of 194 Long Covid studies.^47^

The choice of control group should be appropriate for the research question and counterfactual of interest. For example, control groups in studies of the relationship between COVID-19 vaccination and Long Covid included people with a positive test who had not received a COVID-19 vaccine when infected,^88^, ^89^, ^90^ those with a positive test who had not received a COVID-19 vaccine but were vaccinated against influenza,^91^ and those without evidence of SARS-CoV-2 infection.^92^^,^^93^

A common arrangement in Long Covid prevalence studies was to obtain estimates of current symptomology for people previously infected with SARS-CoV-2 (exposed) and those who have never tested positive or have always tested negative (non-exposed), with Long Covid prevalence then being inferred from the between-group difference. However, this approach became more challenging as the pandemic unfolded, with SARS-CoV-2 infection becoming ubiquitous (over 80% of the population of England may have been infected by November 2022,^94^ and this proportion has since risen) and freely accessible community testing programmes coming to an end. Thus, the pool of potential control participants depleted over time.

An ‘always negative’ control group (i.e. one or more negative tests and no positive tests) may be preferred to a ‘never positive’ one as people who never test, irrespective of symptoms experienced, may be systematically different to those who test regularly in ways related to Long Covid risk, such as sex, ethnicity, and deprivation.^95^ Thus, differential healthcare-seeking behaviours according to these attributes are controlled for by a test-negative design, as is commonly employed in studies of vaccine effectiveness.^96^ These selection effects were mitigated by programmes such as the REACT study^97^ and the COVID-19 Infection Survey (CIS),^98^ which involved testing for SARS-CoV-2 at random, regardless of individuals' symptoms or healthcare-seeking tendencies.

Irrespective of how SARS-CoV-2 test results are obtained, returning a negative test for SARS-CoV-2 is not the same as being infection-free. When evaluated against PCR testing, the sensitivity of lateral flow devices (LFDs) was estimated at 85% among people with high viral concentrations,^99^ but as low as 40% among asymptomatic infections.^100^ The true sensitivity of LFDs is likely to be lower still because PCR tests are also susceptible to false-negative results.^101^ Studies with control groups defined by serological tests are particularly susceptible to bias, because the likelihood of experiencing Long Covid is associated with a weak antibody response to infection.^102^ Any control group defined by negative tests will therefore inevitably be contaminated by people who have had COVID-19, and this exposure misclassification will attenuate the difference in symptom prevalence between the exposed and control groups towards the null of no difference.

Some retrospective studies have made use of historical, pre-pandemic controls.^92^^,^^103^ With this design, the researcher need not worry about the depleting pool of non-exposed participants throughout the pandemic. However, comparisons between the exposed and non-exposed groups are fully confounded by temporal effects. For example, a retrospective study using electronic health records EHRs may be affected by differences in the pre- and during-pandemic availability and delivery of healthcare services.

## Choice of study population

The risk of developing Long Covid varies across socio-demographic groups,^57^^,^^64^^,^^104^ hence study populations with differing socio-demographic profiles will give rise to different Long Covid prevalence estimates. The most obvious differences are between children and adults, and between people who were and were not hospitalised with acute COVID-19. Over 3% of the UK population aged over 16 years self-reported Long Covid in March 2023, compared with 0.5% of children aged 2–16 years.^65^ A meta-analysis found that over 50% of people who were hospitalised with acute COVID-19 experienced persistent symptoms, compared with less than 35% of non-hospitalised cases.^47^ Long Covid prevalence estimates are also associated with the proportion of study participants who were admitted to intensive care or required ventilation.^105^ Prevalence estimates in these groups may be subject to survivorship bias, given the high mortality among patients admitted to hospital with COVID-19 (during the first wave of the pandemic, over 30% of these patients died in hospital^106^); that is, the Long Covid prevalence estimate might have been higher had these patients remained alive in the follow-up period.

Acute-phase symptomatology is predictive of subsequent Long Covid risk.^107^ Using CIS data, the estimated prevalence of self-reported Long Covid 12 weeks post-infection increased from 11.7% to 17.7% when the study population was restricted to participants who were symptomatic at infection.^80^ The denominator is important when interpreting study findings: do estimates relate to the likelihood of developing Long Covid out of *all people infected with SARS-CoV-2*, or only out of *those who exhibited symptoms at the acute phase*? This has implications for generalisability: up to 40% of infections in the first year of the pandemic may have been asymptomatic,^108^ and this proportion is likely to have increased following the emergence of the Omicron variant.^109^

Representative data on asymptomatic infections can only be obtained from random testing programmes such as the REACT^97^ and CIS^98^ studies. Routinely collected data from national testing programmes and healthcare interactions will primarily include symptomatic infections. While some asymptomatic infections may also be included, these are likely to be concentrated in specific, potentially non-generalisable, population subgroups such as healthcare workers who were compelled to test regularly.

Other key determinants of Long Covid prevalence are vaccination status when infected, the SARS-CoV-2 variant of infection, and past infection history. Multiple studies have demonstrated that COVID-19 vaccination is associated with lower odds of developing long-term symptoms after infection.^110^^,^^111^ The Omicron variant was widely reported to result in less severe disease than the previously dominant Delta variant,^112^, ^113^, ^114^, ^115^ and while this appears to be broadly true for Long Covid risk, there is evidence of substantial heterogeneity by COVID-19 vaccination status.^116^^,^^117^ Among people infected with the Omicron variant, the risk of developing new-onset Long Covid symptoms appears to be lower after reinfection than after the first infection.^118^ Less is known about the impact on Long Covid risk of exposure to therapeutics during the acute phase of infection, which could be achieved with long-term follow-up of participants in clinical trials such as RECOVERY.^119^

## Accounting for time-varying factors

**Fig. 2:**
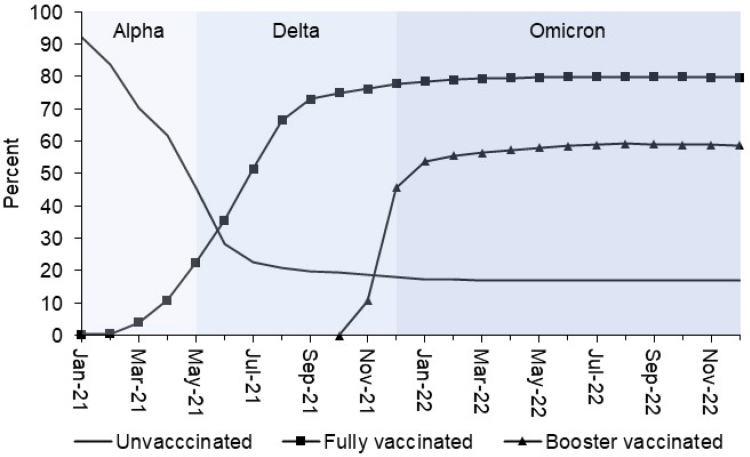
Dominant SARS-CoV-2 variant in circulation in the UK, and COVID-19 vaccine uptake among people aged under 50 years in England, January 2021 to December 2022. The figure has been produced by the authors using publicly available data from the Office for National Statistics, 2023^120^ (periods of SARS-CoV-2 variant dominance); and the Office for Health Improvement and Disparities, 2023^121^ (COVID-19 vaccine uptake data). Mass vaccination began in December 2020 for people aged ≥80 years and those who were clinically extremely vulnerable, but not until April 2021 for people aged under 50 years without underlying health conditions. The majority of fully vaccinated individuals received their second dose 12 weeks after their first, hence most people in the general population started to be fully vaccinated from July 2021 onwards. This coincided with Delta becoming the dominant SARS-CoV-2 variant in circulation in the UK from May 2021, before which the Alpha variant had been dominant.^120^ Little over a fifth of the population of England aged under 50 years had been fully vaccinated against COVID-19 at this time.^121^ In a similar vein, booster vaccine doses were made available to individuals aged under 50 years without underlying health conditions from November 2021, which almost exactly coincided with Omicron becoming the dominant variant in December 2021.^120^ This temporal collinearity between COVID-19 vaccination status and probable SARS-CoV-2 variant of infection, which will be a near-perfect correlation after conditioning on age and underlying health status, renders it practically impossible to eliminate the variant as a potential confounder of the relationship between vaccination status and Long Covid risk.

Over the course of the selected study period, it may be important to control for time-varying factors that affect the epidemiology of COVID-19 and Long Covid, including the rollout of mass vaccination, successive dominance of different SARS-CoV-2 variants, changing knowledge of the virus and disease, or seasonal effects. These may be confounders of the relationship between infection and Long Covid, other exposures of interest and Long Covid, or Long Covid and its downstream impacts; but in practice, near-perfect temporal collinearity may render it impossible to fully control for these factors, as illustrated in Fig. 2. This limitation was noted in one study on vaccine effectiveness in preventing Long Covid, in which 98.9% of the vaccinated group were infected during the Delta period while 99.7% of the unvaccinated group were infected during the Alpha period.^88^ However, the opposite is also true: studies on SARS-CoV-2 variant as a risk factor for Long Covid were temporally confounded by COVID-19 vaccination status.^122^

Besides SARS-CoV-2 variant and COVID-19 vaccination status, other relevant time-varying factors might include the introduction and easing of social restrictions (which were not always geographically homogenous) and increasing public awareness of Long Covid as a phenomenon. While it may not be possible to measure and control for these specific factors, researchers may instead control for calendar time as a “catch-all” approach; for example, several studies have modelled the outcome as a smooth function of the calendar date of infection.^3^^,^^118^

## Electronic health records (EHRs) versus sample survey data

EHR studies may define outcomes based on the presence of diagnostic and referral codes for Long Covid in the patient's health record, or identify clusters of coded symptoms. EHR databases are typically very large, covering a substantial proportion of the relevant population and therefore producing epidemiological estimates of Long Covid with high precision. However, the challenges described below mean that these estimates may be precisely wrong, an example of the so-called “big data paradox”.^123^

Uptake of new diagnostic codes by GPs may have been delayed due to a lack of awareness and differences in coding practices between healthcare providers.^124^ Following the increasing usage of Long Covid codes in primary care through 2021, the trend reversed from the start of 2022.^41^ In one study, only 5% of people with self-reported Long Covid in population-based surveys had a code related to Long Covid in their primary care records.^125^ Some patients may experience difficulties in receiving a diagnosis and accessing services,^126^ and certain population subgroups may be particularly underrepresented in EHR data. For example, there are established gradients in healthcare access by factors such as age, ethnicity and socioeconomic status that predate the COVID-19 pandemic.^127^

The clinical requirement to exclude other aetiologies to diagnose Long Covid means that coded cases in EHRs will have high positive predictive value (the proportion of diagnosed patients who really have Long Covid), they will suffer from low sensitivity (the proportion of people in the population with Long Covid who have a recorded diagnosis). Even if individual symptoms rather than Long Covid diagnoses are used to identify chronic illness following SARS-CoV-2 infection, the symptoms coded in primary care records are unlikely to capture the true symptomatology experienced by individuals with Long Covid, with much symptom-related information instead being held in free-text entries.^64^ This propensity to under-capture people with Long Covid means EHRs cannot be used to estimate prevalence (though they may have utility for comparing long-term symptomology in people previously infected versus not infected with SARS-CoV-2, or before versus after SARS-CoV-2 infection).

Conditional on symptoms, the likelihood of receiving a positive test for SARS-CoV-2 via community testing programmes or in clinical settings, and thus having a positive test result recorded in EHRs, may depend on factors such as age, sex, ethnicity, deprivation, and calendar period of infection.^43^^,^^95^ These differences in test-seeking propensities between socio-demographic groups may reflect between-group heterogeneity and temporal changes in the relative cost of self-isolation after receiving a positive test, the ability to access tests, and awareness of relevant public health messaging. Records from national testing programmes and clinical settings are also likely to under-count asymptomatic infections, which were estimated to comprise nearly half of all infections in some months during the pandemic.^128^ Socio-demographic characteristics, calendar period of infection and acute-phase illness severity are known to be prognostic of Long Covid,^59^^,^^104^^,^^107^ thus inferences obtained from studies using positive tests for SARS-CoV-2 recorded in EHR databases to define the study population may be distorted.

Sample survey data come with their own limitations and biases. In particular, response bias may be present in survey-based Long Covid research, especially for face-to-face data collection, due to the perceived stigma associated with the condition.^129^ Online respondents may also have more capacity to research their symptoms and gain an understanding of Long Covid compared with those being interviewed in person. These factors may have contributed to apparent mode effects seen in official Long Covid prevalence data in the UK,^130^ whereby the likelihood of self-reporting Long Covid was 30% higher after data collection switched from face-to-face to online. Thus, estimates of Long Covid prevalence are likely to be somewhat dependent on the data collection methodology.

Though relatively costly and logistically challenging, collecting data via probability samples (where participants are randomly selected from a pre-defined frame) coupled with appropriate statistical adjustments/weighting will protect against selection bias. Conversely, non-probability samples will almost certainly result in some degree of selection bias. This includes studies based on convenience samples of self-selecting participants recruited via social media campaigns, which were common early in the pandemic.^48^^,^^54^^,^^131^ Selection effects may be especially strong when Long Covid is the main or sole focus of a study, in which people with the disease may feel more motivated to enrol than those without.

Non-response bias may be present in estimates from survey sample data if responders systematically differ from non-responders in ways related to the variable of interest. Although low response rates do not necessarily indicate high non-response bias, they do suggest substantial *potential* for bias. The enrolment rate for the CIS fell to just 12% of invited households by the end of recruitment.^132^ Long Covid prevalence estimates from REACT were based on response rates under 30%,^57^ while those from 10 UK longitudinal studies ranged upwards from 12%.^104^ The CLoCk study achieved a response rate of 13% for its estimates of Long Covid in children and young people.^133^ These seemingly low response rates are not an artefact of the subject matter and are typical of contemporary population-based studies with random sampling. For example, the UK Labour Force Survey, which produces key national economic indicators such as employment and unemployment, had a recruitment rate of 33% at the start of 2024, falling to just 8% among people completing their final follow-up survey one year post-enrolment.^134^

## Recommendations for future epidemiological research into post-infectious sequelae

Long-term health consequences following an acute infection are not unique to SARS-CoV-2, and in the event of future epidemics/pandemics, we should anticipate that a proportion of cases will result in chronic illness. The COVID-19 pandemic provided an opportunity to learn and reinforce important methodological lessons for conducting fast-paced, yet high-quality, epidemiological research into emerging PAISs, as described in Box 1. Such research also has the potential to shed light on pre-existing but historically under-researched PAISs. Indeed, a review of conditions including post-Ebola syndrome, post-dengue fatigue syndrome, post-chikungunya disease, and chronic illness following West Nile virus highlighted that “the overlap of symptoms, signs, and general features of the individual PAISs suggests the involvement of shared pathological pathways and the possibility that common diagnostic markers, or even a unified etiological model, might be established”.^4^ The review also hypothesised several possible pathological mechanisms for pre-existing PAISs that are virtually identical to those that are believed to underpin Long Covid: pathogen reservoir or remnants; autoimmunity; dormant virus reactivation; and multi-organ tissue damage.^4^ Of course, the extent to which Long Covid will share a common pathogenesis and symptomology with future novel PAISs, and therefore the degree to which the implications of our review can be generalised to these PAISs, is unknown.

Box 1 **Implications for future research.**

• **Anticipate that chronic disease and sequelae will probably follow acute infection with a novel pathogen in a proportion of cases.** The presence of post-acute sequelae is not unique to SARS-CoV-2. The capacity for long-term follow-up should be on the research agenda and built into study designs and data collection tools from the outset.
• **Involve people with lived experience in research.** Inclusive Patient and Public Involvement and Engagement (PPIE) should be incorporated at every stage of the study, from conceptualisation and design through to reporting.
• **Define the disease as a priority.** Ideally, this would mean early development of a biological diagnostic test. In the absence of such a test, there is a need for internationally agreed and harmonised epidemiological definitions and data collection tools, to facilitate more robust spatiotemporal comparisons and evidence synthesis.
• **Timely implementation and adoption of diagnostic and referral codes in healthcare systems, and data linkage between systems.** Observational research using EHR databases with near-whole population coverage relies on the existence of disease-specific codes and their routine use by medical practitioners. Even then, these databases will only have utility for robust, unbiased epidemiological research if patients affected by the condition of interest are able to receive a diagnosis and access relevant services, let alone have their diagnosis and referral coded in EHRs. Record linkage between EHR databases, and between these databases and population-based cohort studies, may also facilitate large-scale studies into the real-world effectiveness of therapeutics (e.g. vaccines or acute-phase treatments) in reducing the risk of chronic illness.
• **Invest in population-based studies with random selection from pre-defined sampling frames and routine biological testing.** Such studies are expensive and resource-intensive to operationalise, but this is the only way to guard against selection-induced bias, for example differential test- and healthcare-seeking behaviours inherent in some observational datasets.
• **More prospective studies with high-frequency, longitudinal data collection.** Frequent (e.g. daily) symptom reporting—before and after infection—means that researchers are more likely to identify temporal symptom clusters and characterise cycles of remission and relapse. Prospective, longitudinal data collection also reduces the need for retrospective recall. However, there is a trade-off to consider here: longitudinal data collection places a burden on respondents, and data quality and response rates may suffer as a result, potentially introducing bias and reducing generalisability.
• **Incorporate appropriate control groups into study designs.** It is important to anticipate that the pool of non-infected individuals might disappear reasonably quickly through time and acknowledge that reinfection is possible. There may therefore be a limited window for selecting and utilising controls, so researchers need to maximise the size and follow-up of this group while the opportunity exists.
• **Reflect study sample heterogeneity in the analysis and reporting.** Demographic attributes, measures of acute disease severity, and time-varying characteristics (such as vaccination status, viral variants, and past infection history) are all potential effect-modifiers, and should be considered as such in the analysis. Researchers should clearly define their choice of study and target populations when disseminating findings. They should also consider the potential implications of the study population choice for the estimates and their generalisability to the target population. Additionally, researchers should stratify their analyses by these effect-modifiers if the study population spans multiple groups.
• **Re-purpose existing outcome sets.** A number of patient-reported outcome measures have been developed and validated for Long Covid. Given that we may expect any future pandemics to have multisystem, multidomain impacts, we have an opportunity to embed these existing measures into research infrastructure to allow rapid deployment.
• **More consistent, ready-to-use data.** To understand the impact of an infectious disease and the corresponding post-acute sequelae, standardised data needs to be captured before, during and after any future pandemic. This highlights the importance of continuing existing large-scale data collections, such as birth cohort studies and community cohorts of seasonal respiratory infections. Dormant cohorts in a maintenance-only state, which could be rapidly stood-up in the event of new epidemic or pandemic (sometimes known as “hibernating studies”), may also be considered.

Quantifying the health economic effects of PAISs is necessary to fully understand their burden and to allow use of comparisons and standardised tools for evaluating population health impacts and cost-effectiveness of interventions. In addition to prevalence and risk factor analysis, this could include estimating effects on health-related quality-of-life^135^ or measures of healthcare use.^136^ Accordingly, our recommendations are applicable to research into healthcare provision and the effectiveness of treatments for Long Covid and other PAISs. Firstly, understanding the burden of Long Covid (or other PAISs) on individuals and health systems can directly inform the scale of healthcare provision needed, enabling planning and delivery of appropriate healthcare services. Secondly, epidemiological data should be used to document, monitor and address gaps and inequalities in healthcare provision for Long Covid (or other PAISs).^137^ Thirdly, epidemiological results obtained from observational data can inform potential clinical trials of therapeutics for Long Covid or provide direct evidence of real-world treatment effectiveness, e.g. via the “target trial emulation” framework.^138^

Individuals with Long Covid have been proactive in engaging with and leading epidemiological studies,^36^ including initiating and publishing their own research as part of an international boom in citizen science throughout the pandemic.^139^ Indeed, people with lived experience are a critical component of epidemiological research. Lived experience and other public involvement is invaluable for shaping study designs, developing survey questions and data collection tools, and contextualising and disseminating findings,^140^ and therefore needs to be embedded from the start including pandemic preparatory work.

The success of future studies will be dependent on timely consensus around a single working definition of the disease, something that still remains lacking for Long Covid, while validated Long Covid patient-reported outcome measures may be re-purposed. Diagnostic and referral codes for use in healthcare systems need to be implemented and adopted at pace, not solely to improve patient care, but also to facilitate high-quality observational research using EHRs.

Research into Long Covid has highlighted the importance of prospective study designs incorporating appropriate control groups and longitudinal data collection, and has demonstrated the value of population-based studies with random participant sampling and routine biological testing. Studies such as these are essential for robustly estimating the prevalence of PAISs, and they will need to be rapidly initiated when faced with a future public health emergency involving a novel pathogen.

## Outstanding questions

Despite a proliferation of research into Long Covid during and since the COVID-19 pandemic, several notable questions remain unanswered. Many of these evidence gaps also apply to PAISs more broadly, which have historically been under-researched.

Clearly people experiencing the debilitating symptoms of Long Covid and other PAISs urgently require robustly validated and widely accessible diagnostic and therapeutic options. This will only be achieved by ongoing and intensified efforts to identify and confirm the pathological mechanisms underpinning the disease. But from a purely epidemiological perspective, perhaps most importantly, there remains an open question as to how best to define PAISs for research purposes. Reaching a harmonised working definition with broad international consensus would increase comparability between studies and would likely increase the scientific legitimacy of the research outputs.

Recent epidemiological studies of Long Covid have included three or more years of follow-up,^141^ but given the chronic nature of PAISs, evidence is needed on the natural history and recovery trajectory over several decades, or even the entire life course starting in childhood. There is also a need for greater understanding of the long-term consequences of PAISs at a macro level, such as the impacts on national and international health and social inequalities, and on economic performance.

Most epidemiological studies of PAISs have focussed on the sequalae of a first index infection. While some evidence exists in relation to a first reinfection event,^118^ the impact of repeated reinfections on the risk of new-onset chronic disease and worsening of pre-existing symptoms is unknown. This may be particularly pertinent to Long Covid as SARS-CoV-2 is now in wide circulation throughout the global population, hence individuals may expect to be infected several times over their lifetime.

Much of the research into Long Covid and other PAISs to date has focussed on the individuals experiencing the symptoms. However, there has been relatively little attention paid to the family and household members of these individuals, despite the potential for the existence of such secondary effects. For example, people with Long Covid are known to experience psychological sequalae such as symptoms of depression and anxiety,^142^^,^^143^ and there is evidence that such symptoms may cluster within households and wider social networks.^144^

## Conclusion

Reliable insights into the epidemiology of Long Covid were essential for informing the public health response during the COVID-19 pandemic, and they continue to be needed to inform public service provision and spending decisions as society and the economy recover from the pandemic. However, understanding has been hampered by the wide range of estimates produced across research and surveillance studies. These differences are likely to stem from heterogeneity in data sources, study designs and statistical methods. In preparation for the public health response to future epidemics and pandemics, this review outlines key epidemiological and statistical considerations and recommendations when designing studies of emerging PAISs, focussing on Long Covid as a case study.

## Contributors

DA wrote the original draft manuscript. All authors reviewed, edited and approved the final manuscript and accept responsibility for the decision to submit for publication.

## Declaration of interests

KK was chair of the ethnicity subgroup of the UK Scientific Advisory Group for Emergencies (SAGE) and a member of SAGE. KK has acted as a consultant, speaker or received grants for investigator-initiated studies for Astra Zeneca, Bayer, Novo Nordisk, Sanofi-Aventis, Servier, Lilly and Merck Sharp & Dohme, Boehringer Ingelheim, Oramed Pharmaceuticals, Pfizer, Roche, Daiichi-Sankyo, Applied Therapeutics, Embecta and Nestle Health Science. HW has received speaker fees from BioNTech. MC has received personal fees from Aparito Ltd, Boehringer Ingelheim, CIS Oncology, Halfloop, ICON, Merck, Pfizer, Shionogi B.V. and Vertex outside the submitted work. MC co-developed the Symptom Burden Questionnaire™-Long COVID. SH has received royalties for commercial licences for the Symptom Burden Questionnaire™-Long COVID. DA and RAE were members of the NHS England Long Covid Taskforce. RAE has acted as a consultant, speaker or received grants for investigator-initiated studies for AstraZeneca, Genentec-Roche, and Moderna. CJA has received grants/contracts from UK Research and Innovation (UKRI)/the Medical Research Council (MRC) and the National Institute for Health and Care Research (NIHR). AB has received grants/contracts from NIHR, the National Institutes of Health (NIH), the European Union (EU) and Alzheimer's Research UK. CB has received grants/contracts from Areteia, AstraZeneca, Chiesi, Genentech, GSK and Regeneron. CB has received consulting fees from Areteia, AstraZeneca, Chiesi, Genentech, GSK, Regeneron Pharmaceuticals, Roche and Sanofi. PE has received grants/contracts from NIHR and UKRI. EH has received grants/contracts from NIHR. SMPP has received grants/contracts from NIHR and UKRI/MRC. RS has received grants/contracts from NIHR and UKRI. TS has received grants/contracts from NIHR and UKRI. TS has received support for attending meetings and/or travel via an NIHR Senior Investigator Fellowship. TS is on the Scientific Board Excellence in Paediatrics and the Scientific Advisory Committee of the European Paediatric Association. HW has received grants/contracts from NIHR and UKRI/MRC. HW has received payment/honoraria for lectures and support for attending meetings and/or travel from BioNTech SE. All other authors declare no competing interests.
